# Successful tactile based visual sensory substitution use functions independently of visual pathway integrity

**DOI:** 10.3389/fnhum.2014.00291

**Published:** 2014-05-13

**Authors:** Vincent K. Lee, Amy C. Nau, Charles Laymon, Kevin C. Chan, Bedda L. Rosario, Chris Fisher

**Affiliations:** ^1^Department of Radiology, University of PittsburghPittsburgh, PA, USA; ^2^Sensory Substitution Laboratory, Department of Ophthalmology, Eye and Ear Institute, University of PittsburghPittsburgh, PA, USA; ^3^Department of Ophthalmology, University of Pittsburgh Medical CenterPittsburgh, PA, USA; ^4^McGowan Institute for Regenerative Medicine, University of PittsburghPittsburgh, PA, USA; ^5^Department of Ophthalmology, Louis J. Fox Center for Vision Restoration, University of PittsburghPittsburgh, PA, USA; ^6^Department of Bioengineering, University of PittsburghPittsburgh, PA, USA; ^7^Center for the Neural Basis of Cognition, University of Pittsburgh–Carnegie Mellon UniversityPittsburgh, PA, USA

**Keywords:** sensory substitution, BrainPort, blindness, visual pathways, diffusion tensor imaging, fractional anisotropy

## Abstract

**Purpose:** Neuronal reorganization after blindness is of critical interest because it has implications for the rational prescription of artificial vision devices. The purpose of this study was to distinguish the microstructural differences between perinatally blind (PB), acquired blind (AB), and normally sighted controls (SCs) and relate these differences to performance on functional tasks using a sensory substitution device (BrainPort).

**Methods:** We enrolled 52 subjects (PB *n* = 11; AB *n* = 35; SC *n* = 6). All subjects spent 15 h undergoing BrainPort device training. Outcomes of light perception, motion, direction, temporal resolution, grating, and acuity were tested at baseline and after training. Twenty-six of the subjects were scanned with a three Tesla MRI scanner for diffusion tensor imaging (DTI), and with a positron emission tomography (PET) scanner for mapping regional brain glucose consumption during sensory substitution function. Non-parametric models were used to analyze fractional anisotropy (FA; a DTI measure of microstructural integrity) of the brain via region-of-interest (ROI) analysis and tract-based spatial statistics (TBSS).

**Results:** At baseline, all subjects performed all tasks at chance level. After training, light perception, time resolution, location and grating acuity tasks improved significantly for all subject groups. ROI and TBSS analyses of FA maps show areas of statistically significant differences (*p* ≤ 0.025) in the bilateral optic radiations and some visual association connections between all three groups. No relationship was found between FA and functional performance with the BrainPort.

**Discussion:** All subjects showed performance improvements using the BrainPort irrespective of nature and duration of blindness. Definite brain areas with significant microstructural integrity changes exist among PB, AB, and NC, and these variations are most pronounced in the visual pathways. However, the use of sensory substitution devices is feasible irrespective of microstructural integrity of the primary visual pathways between the eye and the brain. Therefore, tongue based devices devices may be usable for a broad array of non-sighted patients.

## INTRODUCTION

Although retinal implant chips, sensory substitution devices, or gene therapy can provide a state of ultra-low vision to otherwise blind subjects, being able to generate a signal does not ensure the brain will interpret the stimulus appropriately. The condition of the visual cortex is likely to play a major role in the success of visual restoration. There is neurobehavioral evidence to suggest that cortical reorganization after insult to the visual pathways can affect visual perceptions (Dilks et al., 2007). The reorganization of the visual pathways resulting from vision impairment and loss is likely to have significant clinical implications for the prescription of artificial vision devices (Pan et al., 2007; Park et al., 2007; Ptito et al., 2008; Wang et al., 2013). For example, Park et al. (2007) confirmed that the white matter tracts corresponding to visual areas of the brains of non-sighted patients were less organized than sighted controls (SCs); Li et al. (2013) expanded this finding with a larger number of subjects. Both studies suggest that early visual experience is necessary to develop normal visual networks within the brain, but how the networks atrophy with time and how this affects our ability to restore vision is not yet well understood. A number of studies indicate that some visual information can be provided by retinal implant chips (Benav et al., 2010; da Cruz et al., 2013; Dorn et al., 2013) and sensory substitution devices (Nau et al., 2013; Shachar et al., 2013). However, little information is available as to whether duration of blindness or integrity of neural networks affects functional outcomes with such therapies. Thus, it is not always clear if or when these interventions should be undertaken.

The brains of visually impaired individuals process information differently than sighted persons. In blindness the visual cortex, deprived of normal input from the eyes, begins to process information normally handled by other brain areas such as touch and audition. This is referred to as cross modal plasticity, and this concept has already been verified. For example, several recent animal studies have shown that cross modal interactions can be experimentally induced (Allman et al., 2009; Mundiñano and Martinez- Millan, 2010; Van Brussel et al., 2011; Ye et al., 2012). Human based neuroimaging studies have also evaluated the cross modal interplay between visual and auditory processing centers (Kupers and Ptito, 2013). Building on this idea, there are ongoing attempts to use sensory substitution as a means for relaying visual information through non-visual pathways to assist the blind with interpreting their environment (Bach-y-Rita, 1967; Johnson and Higgins, 2006; Hub et al., 2008; Renier and DeVolder, 2010; Chebat et al., 2011; Striem-Amit et al., 2012; Connors et al., 2013; Nau et al., 2013). Multiple investigations have shown that the visual cortex of adult, blind humans is receptive to various types of both tactile and auditory stimuli (Ptito et al., 2005; Merabet et al., 2009; Merabet and Pascual-Leone, 2010). In addition, visual qualia have been evoked in perinatal blind subjects using haptic devices (Ortiz et al., 2011). Studies that have enrolled groups of both early and late blind individuals are somewhat scarce, and more work is needed in this area to understand the mechanisms of cross modal plasticity and how this relates to vision restoration efforts.

The explanation as to how visual experiences can be evoked through non-standard afferent sensory inputs remains controversial. It has been postulated to occur through unmasking of previously dormant connections in the short term (Song et al., 2003) and generation of new connections in the longer term (Gilbert and Wiesel, 1992; Saito et al., 2006). Research also suggests that concurrent sensorimotor experience and training are both likely contributors to adaptive reorganization (Gilbert et al., 2009; Crist et al., 2001; Gilbert and Li, 2012), but the mechanisms underlying sensory substitution in humans have not yet been well defined.

Recent developments in the field of neuroplasticity have shown that the brain is able to undergo experience dependent modifications well beyond a “critical period” of synaptogenesis (Rosa et al., 2013). However, it is not well understood whether sufficient plasticity is retained to restore vision in the putatively atrophied visual pathways of older persons with acquired vision loss.

Because there are scant studies that examine whether duration or etiology of blindness influence cortical receptiveness to cross modal interactions in humans, our ability to make rational decisions about the optimal time after blindness to prescribe artificial vision devices is poor. The purpose of this pilot study was to determine if microstructural integrity of the visual system influenced functional performance of blind subjects using the BrainPort, vision sensory substitution device. Our variables included blindness duration, subject age, and integrity of existing white matter tracts. The results show that the microstructural integrity of acquired blindness falls between those with perinatal blindness and normal vision, and that there was no significant correlation between BrainPort performance and integrity of the visual pathways between the eye and the visual cortex.

## MATERIALS AND METHODS

### SUBJECTS

A total of 52 individuals were enrolled. Participants included 46 completely blind adults (both perinatal and acquired) with a documented visual acuity (VA) of light perception or worse bilaterally, and six normally sighted adults who served as controls. Exclusion criteria included cortical blindness, current smoking, oral lesions or piercings, tongue abnormalities, and any contraindication to neuroimaging. The subjects ranged in age from 22 to 77 years (mean 50 years for blind subjects, and mean age 54 years for SCs) with a gender distribution of 35 men and 17 women. Subjects were recruited from our sensory substitution laboratory research registry that included individuals who initiated contact to participate in artificial vision research studies. Our blind participants had light perception or worse vision bilaterally from a variety of etiologies (**Table 1**). Twenty-six of the above 52 subjects were also enrolled in the imaging arm of this study and had MRI and positron emission tomography (PET) scans. Subjects in the imagining and non-imaging arms were matched to the extent possible for age, gender, etiology, and onset of blindness (rapid versus gradual; **Table 2**).

**Table 1:** Detail of the causes of blindness among our study subjects.

Imaging arm		Non-imaging arm	
Etiology	Subject number	Etiology	Subject number
Trauma	6	Trauma	9
Glaucoma	3	Glaucoma	1
Diabetic retinopathy	2	Diabetic retinopathy	1
Retinopathy of prematurity	3	Retinopathy of prematurity	3
Back surgery	1	Peri-operative	1
Tumor	2	Bilateral retinal vein occlusion	1
Retinitis pigmentosa	1	Temporal arteritis	1
Sepsis	1	Bilateral optic neuropathy	1
Hydrocephalus	1	Congenital	4
Artery occlusion	1		
Meningitis	1		
Congenital	1		

Total	23 + 6 controls	Total	23

*Imaging data for three of the imaging arm subjects were not used due to excessive motion artifact in the scanner. Psychophysical data is pooled for the imaging and non-imaging arms to increase generalizability of results.*

**Table 2:** Exact matching for pathology in studies of bilateral blindness is exceedingly difficult.

Imaging arm		Non-imaging arm	
	Subject number		Subject number
Rapid	11	Rapid	10
Gradual	8	Gradual	5
Perinatal	4	Perinatal	7

*Therefore in addition to age, gender and etiology of blindness, subjects were also matched to the extent possible depending on whether onset of blindness was rapid, gradual or perinatal.*

All subjects gave written informed consent before participation. This research project was approved by the University of Pittsburgh institutional review board and adhered to the tenets of the Declaration of Helsinki. Means and standard deviations were used to calculate the effect size that was needed for the appropriate statistical tests. A total sample size of 26 achieves 80% power to detect an effect size of 0.72 based on a one-way analysis of variance (ANOVA) with significance level of 0.025.

We defined the date of blindness as the year that light perception or worse vision occurred bilaterally. Enrolled study subjects underwent a baseline ophthalmologic exam, including detailed history of the condition leading to blindness, and medical history. Control subjects also underwent baseline ophthalmologic exam to confirm eye health and best corrected vision of at least 20/25 as well as medical history. Affirmations of visual abilities were tested by physical examination and the Freiburg Acuity Test (FrACT; Schulze-Bonsel et al., 2006).

### INTERVENTION

Each study subject underwent an orientation to learn how the BrainPort (Wicab, Inc., Middleton, WI, USA) is worn and operated, followed by five separate, 3 h structured training sessions. Our standard training protocol for basic BP proficiency was developed over the course of 2 years of working with blind patients using the device. The training focuses on the acquisition of core skills, which can be used to achieve more complex tasks. We begin with device familiarity and proceed through exploration of two and three dimensional objects, letter and word recognition, understanding spatial relationships, and mobility (Nau et al., 2013). All training and testing sessions were completed within a 2 week period.

**FIGURE 1:**
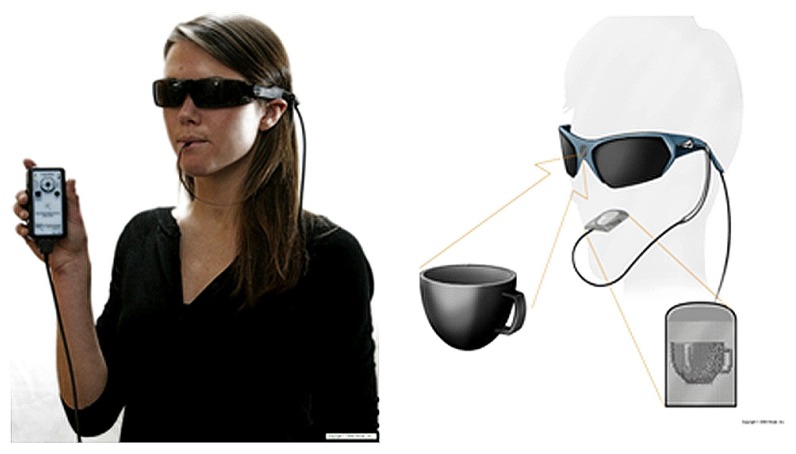
**The BrainPort Vision Device is a sensory substitution system that down samples video from a glasses-mounted frame camera to a 400 pixel (20 × 20) electro tactile array.** Users are able to interpret their environment by feeling the stimuli on the surface of their tongue (Courtesy, Wicab, Inc.).

We employed a prospective, single center, unmasked, within-subjects repeated measures design in which each participant’s functional vision was assessed using a battery of computerized vision tests. Testing was performed at baseline and again after training with the BrainPort Vision device (**Figure 1**).

Experimental use of the BrainPort device was strictly controlled. All subjects included in this study used the device within the laboratory environment, and underwent the same training protocol, administered by the same research staff. None of the subjects were exposed to the outcomes assessment tests described below other than at baseline and the conclusion of the training sequence.

### FUNCTIONAL OUTCOMES

A battery of computer-based tests developed as quantitative examination tools for very low vision assessment was chosen to evaluate visual function, specifically the Basic Light and Motion Test (BaLM) for light perception, time resolution, light localization and motion detection (Bach et al., 2010), the Basic Grating Acuity Test (BaGA) for grating acuity (Wilke et al., 2008), and the Freiburg Acuity and Contrast Test (FrACT) for resolution acuity (Bach, 1996, 2007). Tests were performed according to the standard, published methods developed by our laboratory (Nau et al., 2013).

#### Microstructural diffusion tensor magnetic resonance imaging (DTI)

Twenty six subjects were scanned on a whole body Trio-Tim 3 Tesla MRI scanner (Siemens AG, Erlangen, Germany). The cohort was divided into three subgroups: Healthy normally sighted (NS) Controls (*n* = 6), perinatally blind (PB; *n* = 4), and acquired blind (AB; *n* = 16). For each subject, along with standard localization and proton images, a set of diffusion weighted images were acquired in 12 isotropically distributed directions, repeated four times for signal averaging and noise reduction, using an interleaved spin-echo sequence with the following MRI parameters: echo time/repetition time = 93/4800 ms, acquisition matrix size 128 × 128 × 36 slices, acquisition resolution = 1.875 mm × 1.875 mm × 3.2 mm. The strength of diffusion weighting intensity value (*b*), was at 1000 s/mm2.

To study the white matter fiber tract structure of the subjects, fractional anisotropy (FA) maps were generated for each subject from the diffusion tensor imaging (DTI) scans. The processing and analysis of DTI and FA were conducted with the aid of DTI-Studio, MRI-Studio image processing software (Laboratory of Brain Anatomical MRI, Johns Hopkins University, Baltimore, MD, USA), FSL [tract-based spatial statistics (TBSS); Smith et al., 2004, 2006], and MIPAV (Johns Hopkins, Baltimore, MD, USA) software packages.

#### Positron emission tomography imaging

The same 26 subjects received PET scans before and after training with the BrainPort. For each scan, subjects were injected with 15 mCi of (18F) fluorodeoxyglucose (FDG) and then performed several tasks (object recognition with BrainPort alone, with hands alone and with both the BrainPort and hands). Task performance ended approximately 20 min post-injection at which time the subject was assisted in moving onto the scanner bed. The subject’s head was secured to a head holder using a custom thermoplastic face mask to minimize head motion.

A 10-min transmission scan, required for attenuation and scatter correction, was performed using the scanner’s (HR+ ECAT; Siemens) internal 68-Ge rod sources. PET emission data were acquired for each subject in 35-min frames commencing 60 min post injection.

Positron emission tomography images [128 × 128 × 63 (axial) voxels with voxel dimensions 0.21 cm × 0.21 cm × 0.24 cm] were produced from the raw PET data via Fourier rebinning (FORE) followed by application of the Direct Fourier reconstruction method. Images were smoothed with a 3 mm Hann filter. Reconstruction was performed using the manufacturer’s software and included corrections for attenuation, scatter, accidental coincidences, dead time, and radioactive decay. The three time frames of PET data from each scan session were examined for interframe motion. If necessary, frames were aligned using the software package AIR (Woods et al., 1992, 1998). The aligned frames were summed into a single 15-min image. Each subject’s PET scans were registered to the corresponding pre training anatomical T1 MRI scan.

### DATA ANALYSIS

#### Analysis of functional outcomes and statistical approach

The success threshold for identifying the stimulus correctly in the light perception, time resolution, location perception, motion perception, and grating acuity tests was established as halfway between chance rate (1/number of response alternatives) and 100% correct. Thus, a subject achieved “success” on a particular test when their percent of correct responses was at least halfway between chance rate and a perfect score. This success threshold corresponds to rates for two-alternative forced-choice method (2-AFC), 4-AFC, and 8-AFC at 75.0, 62.5, and 56.25% respectively. This criterion for 24 trials results in the probability of exceeding the threshold by chance at 1.1, 0.011, and 0.000013 percent for 2-AFC, 4-AFC, and 8-AFC. All statistical analyses were two-sided and the significance level was set to 0.05. Data were analyzed using the IBM SPSS Statistics Version 20 data management program. Shapiro–Wilkes Tests of normality were conducted for all outcomes measures. For the psychophysical results, all outcomes except for motion detection were not normally distributed. The non-parametric Mann–Whitney (M–W) test was used for analysis of functional outcomes. All statistical analyses were two-sided and the significance level was set to 0.05.

#### Neuroimaging analysis and statistical approach

Both region-of-interest (ROI) based and voxel-wise approaches were adopted in analyzing the white and gray matter structures. A multi-pronged approach was justified because changes that might occur due to differing etiologies and durations of vision loss are nearly impossible to control for in studies involving blind subjects, and compound the already existing, normal inter-individual differences between subjects. Using several methods to determine ROI and analyzing each in turn allowed us to reduce these confounds and examine consistency of results.

**FIGURE 2:**
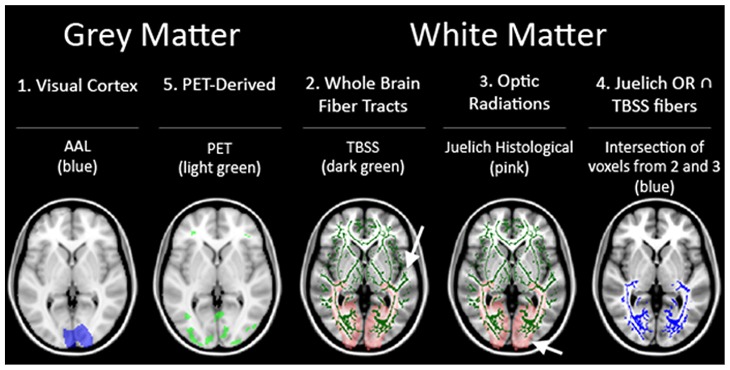
**Summary of the five ROI used in our analysis**.

Each of the subjects’ anatomical scans were registered to the Montreal Neurological Institute’s (MNI) IPBM152 brain template (Mazziotta et al., 2001a,b) and transformations applied to the co-registered FA images. As a result of this process, the 26 FA maps of the controls and blind subjects were transformed to a single unified reference frame within MNI-template based coordinate system (coordinate system/MNI-template based coordinates). This allowed for direct comparisons between the images. Several ROI’s were considered for use in the analysis **Figure 2**.

First, to assess the region of the visual cortex, a mask of the regions corresponding to the visual cortex from the automated anatomical labeling (AAL) for the MNIT152 template was used (ROI 1; Tzourio-Mazoyer et al., 2002). Next, the fiber tracts generated by TBSS (Smith et al., 2006) from the mean FA skeleton served as an ROI representing whole brain fiber tracts not specific to the visual pathways (ROI 2). This ROI was used as a negative control, and served to assess whether any variance uncovered in the other ROIs were due to region-specific significant differences or were caused by heterogeneity of brain structure between individuals regardless of sightedness. Then, a third visual pathway specific ROI was generated from the optic radiations (OR) in the Juelich Histological Atlas for the MNI152 template in FSL software (ROI 3). In addition, a fourth ROI representing the relevant fiber bundles in the visual pathway was generated by taking just the voxels present in the two previous ROI’s [areas of the brain considered to be in both the whole brain fiber tracts (ROI 2) and the Juelich OR’s (ROI3)] (ROI 4). The final set of ROI’s was derived from the PET data (ROI 5). The rationale for including a PET based ROI was to gain insight into whether regions of the brain that were functionally activated by BrainPort use would also show microstructural integrity differences.

To generate the PET derived ROI, each subject’s PET scan (aligned with the subject’s MRI) was warped to match the MNI template using the subject’s MRI-to-MNI transformation determined as described above. A voxel-wise flexible factorial design analysis (between-subject: group and within-subject: condition) was performed in SPM8 (http://www.fil.ion.ucl.ac.uk/spm) to determine the condition effect and the interaction effect between group and condition. The flexible factorial analysis was chosen due to the fact that there were two scans (baseline and post-BrainPort training) performed for each subject. The analysis included three factors [subject (subject number), group (normal SCs, PB, and AB), and condition (pre- and post- training)], and an interaction between group and condition. All PET scans were rescaled within SPM 8 to have the same mean value within the brain. A one-way ANOVA was performed by averaging the conditions across subjects to assess the group effect. The statistical threshold for significance was set to *p* = 0.05 corrected for multiple comparisons [family-wise error (FWE) corrected] and a spatial extent threshold of 27 voxels was required. Thus, these regions were used as the PET-based ROIs.

In summary, there were a total of five ROI’s used as outlined in **Figure 2**. Three white matter only ROI’s consisting of: (ROI 2) whole brain fiber tracts generated by TBSS, (ROI 3) OR from JHA, and (ROI 4) fiber tracts found in both whole brain fibers and JHA OR. The other two ROI’s (ROI 1 and ROI 5) include both gray and white matter.

For each of the chosen ROI’s, descriptive statistics of the mean FA intensity within the region for each experimental group were computed. Examination of normal distribution assumption was determined by q-q plots and histograms. Kruskal–Wallis (K–W) test was performed to determine differences between the three cohorts for non-normal distributed continuous data. *Post hoc* comparisons for the non-normal continuous data were performed using the non-parametric M–W tests and adjustment for multiple comparisons with Bonferroni correction method. Adjusted *p*-values for *post hoc* comparisons are presented. All statistical analyses were two-sided and the significance level was set to 0.05. Data were analyzed using the IBM SPSS Statistics Version 20 data management program.

In the voxel-wise approach, analysis of variance of homologous voxels between the three cohorts was performed to identify areas that might show statistically significant differences. An initial analysis of the FA images was conducted using TBSS, a non-linear registration and alignment-invariant voxel-wise statistical analysis method available as part of FSL software package (Kings-Smith et al., 1994). At the end of the TBSS process, FA images projected onto an alignment-invariant mean FA-skeleton of all 26 subjects were obtained.

The TBSS method has proven to be alignment-invariant and robust in registration and spatial normalization. However, to address concerns about possible effects on the FA analysis due to variations in brain structure, an additional spatial normalization step was carried out. The 26 co-registered FA images, already registered to each other and aligned to the MNI template, were combined to form an average FA image map. From the average FA image map, a mask was created that corrected for any edge misalignments, and variations in ventricular size and border. Through use of threshold erosion, the mask excluded outlier voxels on the borders of ventricles as well as on the outer perimeter of the brain so that the volumes retained for the analysis represent voxels containing neuronal tissue present in all brains.

An initial voxel-wise parametric analysis, part of TBSS, was conducted. However, a *Z*-test on the distribution of the means indicated that PB (*Z* = -3.22) was not normally distributed. Therefore, a non-parametric analysis was performed on the projected, co-registered FA images generated from TBSS using a script custom written in MATLAB to conduct K–W and M–W tests between our subject groups. Analysis of our imaging data were independently verified by authors Vincent K. Lee and Bedda L. Rosario.

## RESULTS

### EFFECTS OF FUNCTIONAL TESTING WITH BRAINPORT

**FIGURE 3:**
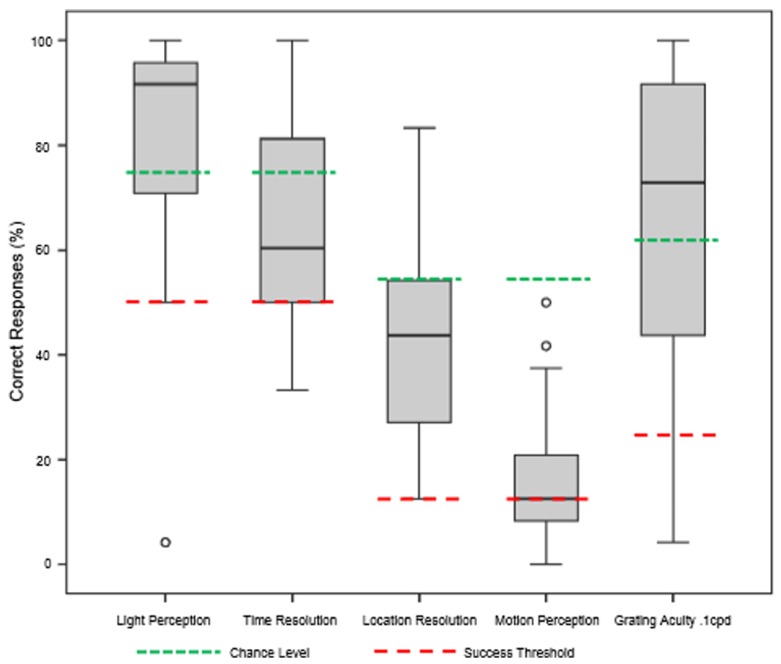
**Summary of functional test results after 1 week of BrainPort training.** Baseline values were all at chance level and are not shown. After training, we are able to demonstrate improvements in performance that reach statistical significance for all tests except motion detection (Wilcoxon signed rank test).

The underlying hypothesis of this study was that visual function would improve with BrainPort use after appropriate training, but the ability to use the device might depend on the duration of blindness. Our results showed that all subjects improved in all six vision tests after completion of the training protocol (**Figure 3**). For the light perception task, mean subject scores improved from 53.6% correct (range 12.5–95.8%) at baseline to 82.8% correct (range 4.2–100%) post training, an increase of 29.2% (*p* < 0.05). Their ability to detect temporal resolution improved from a baseline mean score of 50.8% correct (range 33.3–91.7%) to 63.9% correct (range 33.3–100%), an increase of 13.1% (*p* < 0.05). The location task required the subject to correctly identify the direction in which the light stimulus was oriented. Performance improved from 12.3% correct at baseline (range 0.0–41.7%) to 42.6% correct after training (range 12.5–83.3%), an increase of 30.3% (*p* < 0.05). For detecting stimulus motion, subjects improved from a mean baseline score of 13.1% correct (range 0.0–7.5%) to 16.3% correct (range 0.0–50.0%) an increase of 3.2% (*p* < 0.05). For the grating acuity test, the subjects also performed better after training with a mean baseline score of 24.5% correct (range 8.3–83.3%) to the mean post-training score of 63.9% (range 4.2–100%), an increase of 39.4% (*p* < 0.05). Improvements for the motion detection test did not reach statistical significance (*p* = 0.134).

Along with rudimentary visual function testing, we evaluated the more complex resolution acuity using the FrACT tumbling *E* test. Average subject scores at baseline defaulted to the test minimum of 2.70 logMAR VA. These improved to a mean post-training score of 2.49 logMAR VA (range 2.04–2.70). Both pre and post training values convert to Snellen scores of less than 20/400. Nevertheless, the post-training logMAR VA was 0.21 log units better than pre training scores (*p* < 0.05 by related-samples Wilcoxon signed rank test). This resolution falls in the “ultra-low vision” category, but subjects were able to discern large letters and words using the BrainPort during training sessions, indicating improved function from baseline (data not shown).

**Table 3** compares the performance measures between our imaging and non-imaging groups. There were no statistically different results in any of our outcomes. The motion task approached significance (*p* = 0.058), but this should be interpreted with caution as subsequent pilot studies have shown this stimulus to be too small to be reliably detected with the BrainPort (data not shown).

**Table 3:** Results comparing participants enrolled in the imaging and non-imaging arms.

	Post training score (%) exc. E acuity (logMAR)		Independent-samples Mann–Whitney *U*-test
	Imaging group	Non-imaging group	Significance
Light perception	85.3	80.5	*p* = 0.366
Time resolution	61.7	68.0	*p* = 0.229
Location resolution	45.3	41.0	*p* = 0.435
Motion perception	19.1	13.5	*p* = 0.058
Grating acuity 0.1 cpd	69.9	62.7	*p* = 0.266
Tumbling E acuity (FRaCT)	2.49	2.46	*p* = 0.352

A statistical analysis of the interactions between subgroups failed to show any correlation between duration of blindness, etiology of blindness, gender, age or onset of blindness with performance on any of our behavioral tests either before or after BrainPort training.

### IMAGING: ROI-BASED ANALYSIS RESULTS

**FIGURE 4:**
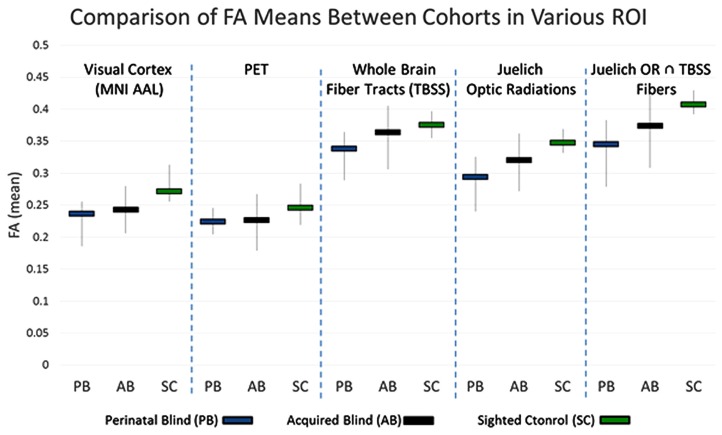
**Range of FA averages within the indicated voxels (vertical lines).** The mean FA from each group is also presented (horizontal bars).

Fractional anisotropy is one method for assessing the differences in the underlying microstructural architecture between subjects. This comparison can be conducted in the whole brain, voxel by voxel. However, in whole brain comparisons, the inherent differences amongst individuals might confound the results and result in false positive or false negative findings. For the five ROI’s discussed in the methods section, the mean FA within these regions was calculated for each subject. The subgroup averages for the three cohorts under each ROI are presented in **Figure 4**. The results show that PB FA averages were less than the NS FA averages for all five ROI’s. Examining the results for the White Matter only ROI’s, we find that the AQ group FA values are intermediate between those of the PB and NS. In the ROI that includes both gray matter and white matter, the visual cortex ROI and PET-derived show similar trends.

Using the mean FA values generated for each subject under the different ROI’s, initial analysis for normal distribution was conducted using histograms and normal quintile plots. This examination showed that non-parametric conditions were appropriate for our data (data not shown). K–W test was performed to determine differences between the three cohorts for non-normally distributed continuous data. *Post hoc* comparisons for the non-normal data were performed using the M–W tests and adjustment for multiple comparisons was performed using the Bonferroni correction method. Adjusted *p*-values for *post hoc* comparisons are presented. All statistical analyses were two-sided and the significance level was set to 0.05. The results are presented in **Table 4**.

**Table 4:** Kruskal–Wallis (K–W) analysis of variance and *post hoc* Mann–Whitney (M–W) comparisons.

ROI	Kruskal–Wallis	Mann–Whitney		
	All three cohorts (*H*-statistic)	*Z-scores from pair-wise comparison*		
		PB–NS	PB–AQ	NS–AQ
Visual cortex (AAL-template)	6.53	2.34	0.28	2.29
PET-derived	3.39	1.92	0.38	1.47
Whole brain fiber tracts (TBSS)	3.76	2.13	1.61	0.15
Juelich OR	6.68	2.56*	1.51	1.62
Juelich OR ∩ TBSS fibers	7.16*	2.56*	1.32	1.92

*The *p*-value significance threshold (α), were set at 0.025 for K–W test, and 0.0083 for M–W test K–W analysis of PET-derived and whole brain fiber tracts showed no statistical significance between PB, AQ, and NS groups. Although M–W comparing PB–NS showed a higher difference (*Z* = 1.92 and *Z* = 2.13), these did not meet the threshold for statistical significance.*

As presented in **Table 4**, the K–W (significance threshold set at *p* ≤ 0.025) analysis showed that there was no statistically significant difference amongst the FA-averages between the three cohorts within the PET-derived (*H* = 3.392) and whole brain fiber tract (*H* = 3.766) ROI’s. This result is not unexpected, however, as the whole brain fiber tracts are white matter structures non-specific to the visual cortex, and the pet-derived ROI contains both gray and white matter. However, K–W analysis did show that there were significant differences in mean FA values (*H* = 7.157) between the cohorts for the last ROI. This last ROI, formed from intersection of Juelich OR and fibers derived from TBSS, contain voxels which are the most specific to major white matter fiber tract within the OR. The significant differences in mean FA values among the three cohorts in this major posterior white matter tract along the visual pathway suggest that there may be structural differences between NS, PB, and AQ. The M–W test between PB and NS (*Z* = 2.558) showed that the mean FA differences between these two groups were significant.

The major posterior white matter tracts and Juelich optic-radiation ROIs showed a higher *H*-statistic (*H* = 6.534 and *H* = 6.682, respectively), suggesting that the mean FA-averages within these ROI’s may have a higher difference between the cohorts. However, they cannot be considered statistically significant, and null hypothesis cannot be rejected with the adopted significance threshold which is *H*-critical = 6.975 (*p* < 0.05). It is of note that these two ROI’s which are more specific to the visual pathway, compared to the previous two ROI’s, showed a higher *H*-statistic than whole brain fiber tracts. The higher *H*-statistic suggests that the differences in FA means between the three cohorts increased, which can be seen in graphical form for visual cortex and Juelich OR in **Figure 4** where the FA average for NS is relatively much higher than the averages of the other two cohorts. The M–W tests between PB and NS, and NS and AQ (*Z* = 2.345 and *Z* = 2.285, respectively) for the aforementioned visual cortex ROI supported this observation. The M–W test also showed that within the Juelich OR ROI, the PB and NS are significantly different (*Z* = 2.558, which is above the *Z*-critical = 2.395 for).

A regression analysis was performed between duration of blindness and mean FA of all the ROI’s being studied. Two additional ROI’s were also studied (1) The somatosensory tract (obtained from Juelich template) was used as a control pathway and (2) the whole brain mean FA of each subject was determined and used in the analysis. The results are presented in **Table 5**. Of all the ROI’s, only the visual cortex shows a statistically significant inverse correlation between mean FA and duration of blindness. The analysis excluded the PB subjects since other studies have suggested the reduction in FA is not seen in PB subjects (Wang et al., 2013).

**Table 5:** Regression analysis between duration of blindness and mean FA.

ROI	*R*^2^	Correlation	*p*-value
1	Visual cortex	0.21331334	-0.461858571	0.030471885
5	PET-derived	0.03009144	-0.173468844	0.440097828
2	Whole brain fiber tracts	0.037833967	-0.194509556	0.385720695
3	Juelich OR	0.117863148	-0.343312028	0.117756143
4	Juelich OR ∩ TBSS fibers	0.054816952	-0.234130204	0.294301625
5	Whole brain FA	0.044513063	-0.210981192	0.171704325
6	Somatosensory	0.091303281	-0.302164328	0.345939932

### IMAGING: VOXEL-WISE ANALYSIS RESULTS

**FIGURE 5:**
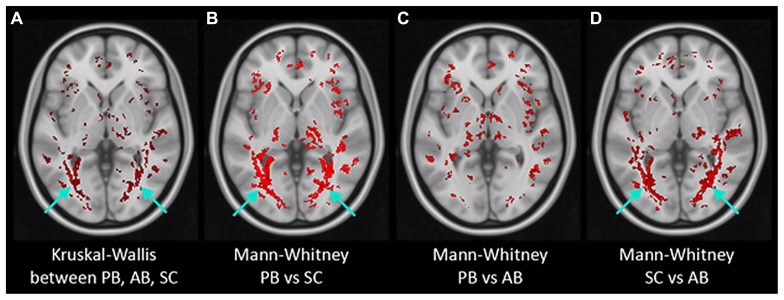
**Results of non-parametric voxel-wise analyses of FA maps.** Voxels with statistically significant differences (in red) were found in the bilateral optic radiation (arrows) in Kruskal–Wallis test of the three groups **(A)** and in Mann–Whitney tests between PB and Normals **(B)** and between AB and Normals **(D)** but not between PB and AB **(C)**.

**Figure 5** presents the results of post-TBSS processing and voxel-wise non-parametric analysis of FA. In (**Figure 5A**) are the results from the K–W analysis, after FWE corrections for multiple comparison, showing voxels (in red) which were statistically significant (*H* critical = 7.38; *p* ≤ 0.025). The voxels showing significant differences between the three cohorts correspond to OR bilaterally (arrows). Results of M–W tests showing voxels with statistically significant *Z*-scores (*Z* critical = 2.40; *p* ≤ 0.00833) between PB and NS, PB and AQ, and NS and AQ are also shown. There were statistically significant voxels in the OR between PB and NS (**Figure 5B**), and between NS and AB (**Figure 5D**), but not between PB and AB (**Figure 5C**) derived ROI (*p* = 0.04).

**FIGURE 6:**
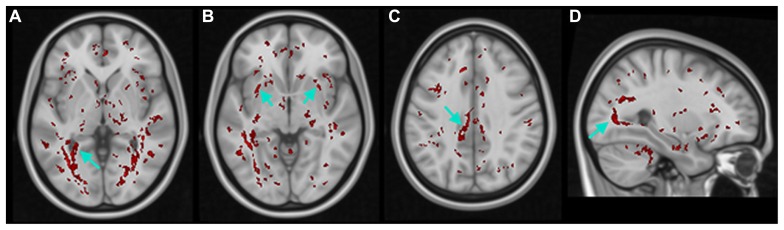
**Results of non-parametric FWE adjusted voxel-wise K–W analyses of FA maps.** Voxels with statistically significant differences (in red) within THE **(A)** lingual gyrus (LG), **(B)** inferior fronto-occipital fasciculus (IFOF), **(C)** cingulate gyrus (CG), and **(D)** inferior longitudinal fasciculus (ILF).

Additionally, in the K–W analysis, statistically significant clusters were uncovered in major tracts or surrounding brain regions that are associated with visual cortex (**Figure 6**, white arrows) such as the lingual gyrus (LG), inferior fronto-occipital fasciculus (IFOF), cingulate gyrus (CG), and inferior longitudinal fasciculus (ILF). These fiber tracts possess indirect connections to the visual cortex (Catani et al., 2002, 2003) and are involved in visual processing such as object recognition, dreaming, and other complex visual functions (Hickey et al., 2010; Ortibus et al., 2012).

No relationship was found between FA and our performance measures with the BrainPort.

## DISCUSSION

Brain plasticity is now believed to continue throughout life, albeit likely at a slower pace than the prolific neural maturation characteristic of childhood. Blindness is an intriguing model to study neuroplasticity in adulthood for several reasons. It is easy to simulate acute blindness in the NS, and it is relatively easy to obtain persons with different durations of acquired blindness. Therefore, adaptive processes can be investigated by comparing cohorts with different life fractions of vision loss. These groups can be compared to cohorts with perinatal blindness, which are the most likely to exhibit extreme structural and functional abnormalities of the visual system. Moreover, the visual cortex and its association areas possess a myriad of connections to other brain areas, lending insight into the microstructural changes that both precede and result from cross modal plasticity in ways that other sensory losses may not access.

Of all the brain regions we explored, only the visual cortex shows a statistically significant inverse correlation between mean FA and duration of blindness. As the duration of blindness increases, FA means go down, indicating decreasing white matter tract integrity. Our finding that the brains of our AB group have FA values that are more similar to those with perinatal blindness than to those with sight carries possible implications for the most appropriate methods and timing that will be employed for vision restoration in the clinical setting. The extent to which the visual cortex is structurally and functionally remodeled after sensory loss is still a matter of controversy. Using a 1.5T MRI scanner, Schoth et al. (2006) failed to show any structural differences in an AB cohort using DTI. Yet other, more recent studies have suggested that white matter tracts within the visual pathways in the AB devolve to a disorganized state over time (Wang et al., 2013). Our findings of decreased white matter tract integrity as a function of blindness duration support this latter conclusion, and may be due to a larger sample size and use of a 3T scanner. Recently projects in the lab of Dr. Chan (data not shown) and others have demonstrated neurodegenerative changes associated with progression of glaucoma using functional imaging studies (Dilks et al., 2007; Pan et al., 2007; Ptito et al., 2008).

The positive correlations between disease severity and cortical changes suggests that the visual brain changes in parallel to ocular pathology. Therefore, it could be argued that vision restoration efforts that rely on traditional visual pathways may confer better results when the duration of blindness is short. For example, stimuli produced by ocular based technologies may have a better chance of being converted into a meaningful precept by a still relatively intact visual processing network. Conversely, when the duration of blindness is longer, the visual brain is likely to have been modified by deafferentation and the experience of being blind. In this setting, technologies which bypass the visual system, such as the BrainPort, could be a better option. However, sensory substitution approaches have their own set of neurological barriers. For example, one hypothetical obstacle to successful SSD use is that inherent multisensory feedforward connections may not be sufficient to enable functional use of alternative pathways, even under ideal circumstances (Gilbert and Li, 2013; Mancuchi and Kurniawan, 2013). However, with long term blindness, this “bandwidth” issue may be mitigated to some extent by training and experience. Indeed, it has been argued that sensory substitution use is predicated on the adaptive reorganization of neurons to integrate the functions of two or more sensory systems (Li et al., 2013). Studies exist which suggest experience dependent modifications in response to events or training after the critical period of neural development can influence visual cortical processing (Halko et al., 2011; Chung, 2012; Tao et al., 2013).

While there is now an abundance of evidence to indicate that neural remodeling can occur well beyond the critical period, it is still not clear to which extent such a reorganization can transpire in an older population that is the most likely to be afflicted with acquired blindness. Nevertheless, all of our adult subjects were able to perform simple tasks indicating improved function over baseline values using the BrainPort device. Our behavioral results do echo prior studies which compared the ability of PB to SCs using a tongue display unit for various simple discrimination tasks (Ptito et al., 2005, 2012; Chebat et al., 2007; Matteau et al., 2010).

Our analysis showed that the selected visual ROI and nearby visual association were the only areas which showed statistically significant white matter tract alterations. We also failed to demonstrate any performance difference between our subject groups. Taken together, this implies that the blind can exploit remaining, intact functional areas to access visual based environmental perceptions through sensory substitution, and that the microstructural integrity of the visual pathways (related to duration of blindness) may not be a paramount concern for this method of restoring some “visual” functionality. However, many questions remain. Future work should examine larger numbers of subjects with short term blindness to determine the time course of structural changes. This would inform decisions in the therapeutic domain about when best to intervene with vision restoration. Proton magnetic resonance spectroscopy has recently been reported as a way to look into the neurochemical changes in the visual cortex in the blind compared to sighted subjects (Bernabeau et al., 2009) and may be a way to mechanistically approach questions regarding cross modal plasticity. Our study spanning only a 2 week period has limited capacity to shed insight on the synaptic modifications that might occur with long term sensory substitution use. Prospective studies with cohorts using SSD over longer periods of time will help to answer this question and also reveal whether the microstructural changes seen in blindness can be reversed with intensive training. Other questions include whether decreased FA resulting from blindness renders the brain more (or less) susceptible to cross modal plasticity, and whether this susceptibility might confer performance advantages in the context of vision restoration with sensory substitution. In our studies of over 100 total participants, subjects with prior experience on the Opticon device, (a tactile based reading instrument for the blind) were able to outperform their peers without this experience, although Braille reading alone did not seem to confer any performance benefit. Ultimately, answers to these questions will be critical for clinical decision making in choosing candidates for visual prosthetics.

This study is limited by small size, due to which comparisons between groups are prone to both Type I (false positive) and Type II (false negative) errors. Although the *p*-values were adjusted for multiple comparisons with appropriate statistical tests, it is possible that significance observed in some comparisons are due to chance (Type I error). Similarly, a lack of significance for some comparisons could have been due to limited power to detect a difference (Type II error). However, there is precedent in the literature for neuroimaging studies of the blind with limited sample sizes (Catani et al., 2003; Schoth et al., 2006; Matteau et al., 2010; Ortibus et al., 2012; Ptito et al., 2012). Regardless, our conclusions should be interpreted with caution and considered preliminary. Another limitation is that all of our study subjects had undergone comprehensive blind skills training as a pre-requisite for being enrolled. Therefore, some experience dependent plasticity may have already occurred in this population.

This study demonstrated statistically significant improvements in psychophysical tests when using a vision sensory substitution device that occurred irrespective of blindness duration, subject age, etiology of vision loss, or microstructural status of the visual pathways and whole brain. We demonstrated the existence of definite brain areas with significant FA differences among perinatal blind, AB, and SC subjects, and showed these differences are most pronounced in the brain areas that are more specific to the visual pathways. Regression analysis shows that the duration of blindness is linked to decreased white matter integrity restricted to visual parts of the brain. The results suggest that tongue based sensory substitution devices might be used by persons with varying etiologies and durations of blindness.

## AUTHOR CONTRIBUTIONS

Vincent K. Lee: assist with fMRI/DTI protocol design, data analysis, statistical support, manuscript preparation, manuscript proofing; Charles Laymon: PET protocol design and data acquisition for PET and MRI, data analysis, statistical support, manuscript preparation, manuscript proofing; Kevin C. Chan: data analysis, manuscript revision and preparation, manuscript proofing; Bedda L. Rosario: data analysis, statistical support, manuscript proofing; Chris Fisher: data acquisition for behavioral outcomes, assistance with PET data acquisition, interpretation of data, manuscript preparation; Amy C. Nau: overall protocol development and design, IRB approvals, funding of project, recruitment of subjects, data acquisition for behavioral outcomes, data analysis, manuscript preparation, manuscript proofing.

## Conflict of Interest Statement

The authors declare that the research was conducted in the absence of any commercial or financial relationships that could be construed as a potential conflict of interest.
